# The impact of transitional care on geriatric rehabilitation outcomes: prevalence and associated factors of sarcopenia and malnutrition

**DOI:** 10.1007/s40520-026-03341-3

**Published:** 2026-03-11

**Authors:** Chiara Vetrano, Eva Ritter, Theresa Wahrstätter, Eva-M. Adamer, Patricia Riedl, Ferdinand Prüfer, Špela Matko, David Riedl, Michael J. Fischer, Vincent Grote, Barbara Strasser

**Affiliations:** 1 Ludwig Boltzmann Institute for Rehabilitation Research, Vienna, Austria; 2 Medical University of Innsbruck, Innsbruck, Austria; 3 Rehabilitation Center Kitzbühel, Kitzbühel, Austria; 4 Short-term Care und Transitional Care, Nursing Home Kitzbühel, Kitzbühel, Austria; 5 Department of Orthopaedics and Trauma, Medical University of Graz, Graz, Austria; 6 Medical Faculty, Sigmund Freud Private University Vienna, Vienna, Austria

**Keywords:** Geriatrics, Transitional care, Sarcopenia, Quality of life, Outcome measurements

## Abstract

**Background and aims:**

Geriatric patients are faced with ongoing care needs after hospitalization. This study evaluated the prevalence of sarcopenia and malnutrition at admission and related clinical characteristics in geriatric inpatients of a transitional care program.

**Methods:**

A cross-sectional study and a longitudinal observational study were conducted among geriatric inpatients in a retirement home. Sarcopenia was assessed using the Sarcopenia Definitions and Outcomes Consortium criteria, and nutritional status via the Mini Nutrition Assessment. Patient- and clinician-reported outcome measures, including the Barthel Index, EQ-5D-5 L, NPRS, HAQ-DI, 10 m walking test and Timed Up and Go test were collected at admission and discharge (mean stay: 39 ± 22 days). In addition, a 3-month post-discharge follow-up telephone interview was conducted.

**Results:**

Out of 72 geriatric rehabilitation inpatients (mean age: 84 ± 7 years, 83% female), the prevalence of sarcopenia was 80.6%, while 81.2% of patients were malnourished or at risk of malnutrition upon admission. Sarcopenic patients showed reduced health-related quality of life (EQ-5D-5 L; *p* < .05, *d* = 0.61), greater hand-grip strength asymmetry (68.6%; *p* < .05), and a trend to have a lower functional ability to perform activities of daily living (HAQ-DI; *p* = .06). The transitional care program improved quality of life, care needs, and mobility (all *p* < .001), independently of sarcopenia or malnutrition status.

**Conclusion:**

This study showed a high prevalence of sarcopenia and malnutrition in geriatric transitional care inpatients. Despite improvements in function and quality of life, persistent sarcopenia underscores the need for ongoing, individualized interventions such as progressive resistance training combined with nutritional support.

## Introduction

Demographic changes resulting in an aging population are challenging healthcare systems worldwide, and new geriatric care models tailored to meeting the unique needs of this patient population are needed. Geriatric rehabilitation plays a key role in alleviating and preventing physical limitations associated with multimorbidity and frailty, which are prevalent in older adults. Multimorbidity, defined as the presence of multiple chronic conditions, increases the risk of functional decline, diminished quality of life, and mortality [1, 2]. Frailty, characterized by reduced physiological reserves, is associated with a greater risk of hospitalization and reduced autonomy [3, 4].

Sarcopenia, a syndrome characterized by low skeletal muscle mass, muscle strength, and/or physical performance, can be considered as one of the main physical drivers of frailty [5]. Over 50% of geriatric rehabilitation patients are estimated to suffer from sarcopenia [6], which is associated with a significantly higher risk of mortality post-discharge from geriatric rehabilitation [7]. Furthermore, malnutrition is highly prevalent in rehabilitative inpatient settings; both conditions frequently co-occur [8, 9] and are linked with worse functional recovery [10–12]. The reduction in physical capacity caused by sarcopenia can increase levels of dependency and disability and may influence patient reported outcome measures (PROMs), such as the health-related quality of life [13].

Geriatric patients are faced with a continuum of care needs after hospitalization, requiring a multi-disciplinary approach that bridges acute care and rehabilitation. Transitional care models have emerged as an important intervention for addressing these needs [14]. In particular, these models have been designed to restore muscle mass and physical function, improve quality of life, and enable patients to live more independently, thereby reducing the risk that they will require long-term care. The rehabilitative transitional care model examined in the present study represents a bridge between Phase I and Phase II of a broader framework including acute care (Phase I), post-acute rehabilitation (Phase II), outpatient rehabilitation (Phase III), and long-term preventive measures (Phase IV), based on the WHO rehabilitation phase classification [15]. This framework provides up to 12 weeks of rehabilitative support after a hospital stay due to acute illness [16].

Mobility impairments critically affect geriatric populations, reducing their independence and societal participation [17]. Standardized assessments such as the timed up and go test (TUG) and the 10-meter walking test (10-MWT) are widely used to evaluate these outcomes [18]. Similarly, care dependency measured by using standardized instruments like the Barthel Index, which provide insights into patients’ abilities to perform basic activities of daily living (ADL) and their self-sufficiency [19]. Health-related quality of life (HRQoL) is another domain. Instruments like the EQ-5D-5 L are commonly used in geriatric populations and reflect multidimensional aspects of the patient’s health status with respect to their physical, psychological, and social well-being [20].

In order to comprehensively evaluate outcomes in geriatric rehabilitation, both PROMs and clinician-reported outcome measures (CROMs) are commonly used in the field of sarcopenia research [21]. PROMs, such as the EQ-5D-5 L health-related quality of life questionnaire and pain scales, enable researchers to determine the patients’ subjective health experiences. Conversely, CROMs, including the TUG and the 10-MWT, provide objective assessments of physical function and mobility. Together, these complementary assessments offer a holistic view of rehabilitation outcomes and can be used to bridge the knowledge gap between subjective perceptions and measurable clinical changes [22, 23]. Both clinical indicators are measures designed to provide data on the functional patient status, providing more detailed information about the course of the rehabilitation [24, 25].

Although of vital importance, the effectiveness of geriatric rehabilitation depends on multiple factors, including the patient’s age, baseline functional status, and length of stay in the program. Studies suggest that earlier intervention in the post-hospitalization period and longer durations of rehabilitation are associated with better mobility and quality of life outcomes [26, 27]. Furthermore, the muscle health status and existence of sarcopenia at admission to geriatric rehabilitation may influence rehabilitation outcomes. For example, patients with sarcopenia had a worse functional status, similar functional improvement during rehabilitation, and a lack of recovery after returning home in a prospective study of 99 patients (average age: 84.6) admitted to a subacute geriatric care unit [28]. While measures of muscle health status at baseline predicted the level of dependence of an older population in terms of their ability to perform ADLs [29], a recent retrospective cohort study in 319 older patients found that a high skeletal muscle mass index (≥ 7.0 kg/m^2^ in men and ≥ 5.7 kg/m^2^ in women) at admission was an independent factor negatively influencing improvements in ADLs in older patients in a convalescent rehabilitation ward [30].

The current study was carried out to evaluate the effectiveness of a multi-professional transitional care program in a retirement home (Kitzbühel, Austria), aimed at improving the functional capacity and quality of life in older persons, and thereby promoting independence in older persons after hospitalization. By contextualizing these findings within the broader framework of geriatric rehabilitation, this program has been designed to improve the collaboration between healthcare providers in the transition from hospitalization to rehabilitation or outpatient care and to contribute to evidence-based approaches that support the physical recovery of older patients after their discharge from hospital.

The primary aim of this study was to provide information about the prevalence of sarcopenia and malnutrition at admission to transitional care and to determine whether this prevalence influences transitional care outcomes. We also considered possible moderating factors like the patient’s age, BMI, and length of stay as well as rehabilitation outcomes for both the prevalence of sarcopenia and malnutrition at admission as well as possible changes in sarcopenia from admission to discharge. The secondary objective was to assess the changes in quality of life, care needs, and mobility from admission to discharge in patients receiving transitional care.

## Methods

### Study design and population

#### Study design and setting

To evaluate the rehabilitative transitional care program, a longitudinal observational study was conducted in 2022/2023 at a retirement home in Kitzbühel, Austria, including 126 patients who had been recently discharged from acute hospital care. The study represents a real-world evaluation of an existing care service. Data were collected at admission and at discharge by incorporating both PROMs and CROMs assessed during routine clinical care. Three months after discharge, patients or their relatives were invited to participate in a brief telephone interview.

#### Study center and transitional care

The study was conducted at the transitional care unit of the Kitzbühel retirement home, which collaborates closely with the local rehabilitation and outpatient therapy center. The unit provides up to 24 beds for patients requiring rehabilitative short-term care after hospital discharge. Care is delivered by taking a multidisciplinary approach that includes physiotherapy, ergotherapy, massage, lymphatic drainage, movement therapy, cognitive therapy, and washing and dressing training. This sums up to 105 to 150 min of therapy per day on average based on the individualized rehabilitation goals. The program is embedded in the regional transitional care agreement with the State of Tyrol and complies with national staffing guidelines.

#### Participants and recruitment

Participants were recruited from March 2022 to July 2023 from a pool of acute-care hospital discharges who required further rehabilitation but were unable to access standard geriatric rehabilitation services due to long waiting lists or their health condition. Eligibility for transitional care is formally defined by admission criteria issued by the Province of Tyrol [31] and was not determined by the study team.

The present study was explicitly designed to evaluate the transitional care program in its intended target population, namely patients who meet these predefined criteria and for whom standard geriatric rehabilitation pathways are not feasible or available and, and who, based on their clinical condition, require continued care beyond the average length of stay in acute care, thus serving as a bridge between acute care and rehabilitation.

Eligible patients were asked to participate in the study within the first two days of their rehabilitation stay and provided their written informed consent after receiving a detailed explanation of the study and its potential risks. The study was designed as a per-protocol, real-world evaluation of an existing transitional care program. As recruitment followed the official admission criteria and official regulations defined by the Province of Tyrol [31], the sample size reflects all available transitional care patients admitted during the recruitment period. Length of stay in the transitional care program was determined by individual recovery progress and care needs.

#### Inclusion and exclusion criteria

Participant eligibility was assessed on the first day of admission by specifically trained healthcare professionals at the transitional care institution. Inclusion criteria were: age ≥ 60 years, Barthel Index > 39, receipt of care allowance, sufficient proficiency in the German language, and the ability to provide their informed consent. Only patients who participated in the program for at least two weeks were included in the study. Subjects were excluded if they required acute medical care, suffered from cognitive impairments or dementia, showed a lack of capacity for improvement in care dependency due to chronic-degenerative conditions or injuries, had a Barthel Index ≤ 39, or were participating in another clinical trial.

### Data collection and measurements

Assessments we report here were conducted at admission and at discharge. A follow-up telephone interview was also carried out to assess the patients’ current living situations and levels of care. Data were collected and managed using the pre-installed electronic system CHES (Computer-based Health Evaluation System [32]), ensuring secure and standardized data handling. Primary outcomes were the sarcopenia prevalence and nutritional status at admission. Secondary outcomes include rehabilitation outcome measurements and respective changes in domains (Table 1) from admission to discharge. From 126 initially assessed patients, only those fulfilling the predefined inclusion criteria with a minimum length of stay and complete PROMs and CROMs data were included in the final analytical samples.

#### Sarcopenia

The presence of sarcopenia was assessed according to the Sarcopenia Definitions and Outcomes Consortium (SDOC) guidelines [33]. The cut-off for handgrip strength (HGS) was < 20 kg for women and < 35.5 kg for men and for gait speed < 0.8 m/s. Applying SDOC criteria, patients were divided into three groups: sarcopenia (low HGS and decreased gait speed), pre-sarcopenia (low HGS or decreased gait speed), and non-sarcopenia (normal HGS and normal gait speed). The maximum HGS (kg) was determined using a hand dynamometer (Saehan Corporation, South Korea). Three trials were performed on each hand with the best hand being used to assess the maximal HGS. Individuals whose dominant or non-dominant hand displayed a HGS > 10% stronger were classified as having dominant or non-dominant HGS asymmetry, respectively; this has been associated with functional disability [34].

#### Nutritional status

The nutrition status of each participant was assessed upon admission using the Mini Nutrition Assessment Long Form (MNA-LF) questionnaire [35, 36]. It involves a screening and a detailed assessment, with scores classifying status. If the participant’s score was 11 or less, indicating a “malnutrition risk”, a trained dietitian continued to ask the remaining questions to obtain additional information about factors that could impact their nutritional status. A score between 17 and 23.5 points indicated that the participant was “at risk of malnutrition”, and a score of less than 17 points indicated that they were “malnourished”.

#### Outcome measurements and domains

PROMs and CROMs were used to assess the effectiveness of transitional care. Outcomes were grouped according to their content domains (mobility, care needs, and quality of life). While the introduction distinguishes between PROMs and CROMs as methodological categories, the present classification reflects the clinical content of the assessed constructs. The mobility domain comprised CROMs, while care needs and quality of life were assessed using PROMs. PROMs included the EQ-5D-5 L questionnaire [37] and the Numeric Pain Rating Scale (NPRS), used to evaluate quality of life and subjective pain [38], respectively. The Barthel Index and the Health Assessment Questionnaire (HAQ-DI) were used to assess the patients’ care needs [39, 40]. CROMs, namely the timed up and go Test (TUG) and the 10 m walking test (10-MWT), provided standardized, objective measures of mobility and physical function in the geriatric population [41, 42]. Sociodemographic data, such as age, gender, BMI, and indication groups, were collected upon admission.

**Table 1:** Outcome measurements and domains

Domain	Outcomes
I. Quality of Life	EQ-5D-5 L
	Numeric Pain Rating Scale (NPRS)
II. Care needs	Barthel Index
	Health Assessment Questionnaire (HAQ-DI)
III. Mobility	Timed up and go test (TUG)
	10-meter walking test (10-MWT)

#### Hypotheses

The following hypothesis were tested:

1. We expect a high prevalence of sarcopenia and malnutrition in transitional geriatric care patients.
2. Sarcopenia is associated with worse physical and psychological functioning.
3. The majority of patients report significant improvements in their quality of life, care needs, and mobility from admission to discharge.
4. The presence of sarcopenia upon admission is associated with the trajectory of rehabilitation outcomes from admission to discharge.

#### Statistical analysis

Data were analyzed according to the per-protocol principle as described in the study protocol (cf. Ethical Approval). The prevalence of sarcopenia and malnutrition at admission was calculated using descriptive statistics. Differences in baseline characteristics were tested using *t*-tests and χ^2^. Changes in outcome measurement domains between admission and discharge were assessed using paired *t*-tests and mixed ANOVAs with time (admission vs. discharge) as within-subject factor and sarcopenia status at admission as the between-subject factor. The domains were formed by z-standardizing for both outcome measurements and domains to ensure comparability. Cohen’s d was calculated for t-tests, and partial Eta squared (η²) for ANOVAs, following conventional thresholds for small (d = 0.2, η² = 0.01), medium (d = 0.5, η² = 0.06), and large (d = 0.8, η² = 0.14) effects [43, 44]. All statistical analyses were performed with SPSS (v29). A significance level of *p* < .05 (two-tailed) was considered as statistically significant.

#### Ethical approval

The study was approved and registered by the Medical Ethics Research Committee of Innsbruck Medical University (approval date: 22.03.2022, EC protocol number: 1026/2022).

## Results

### Participant characteristics

**Fig. 1:**
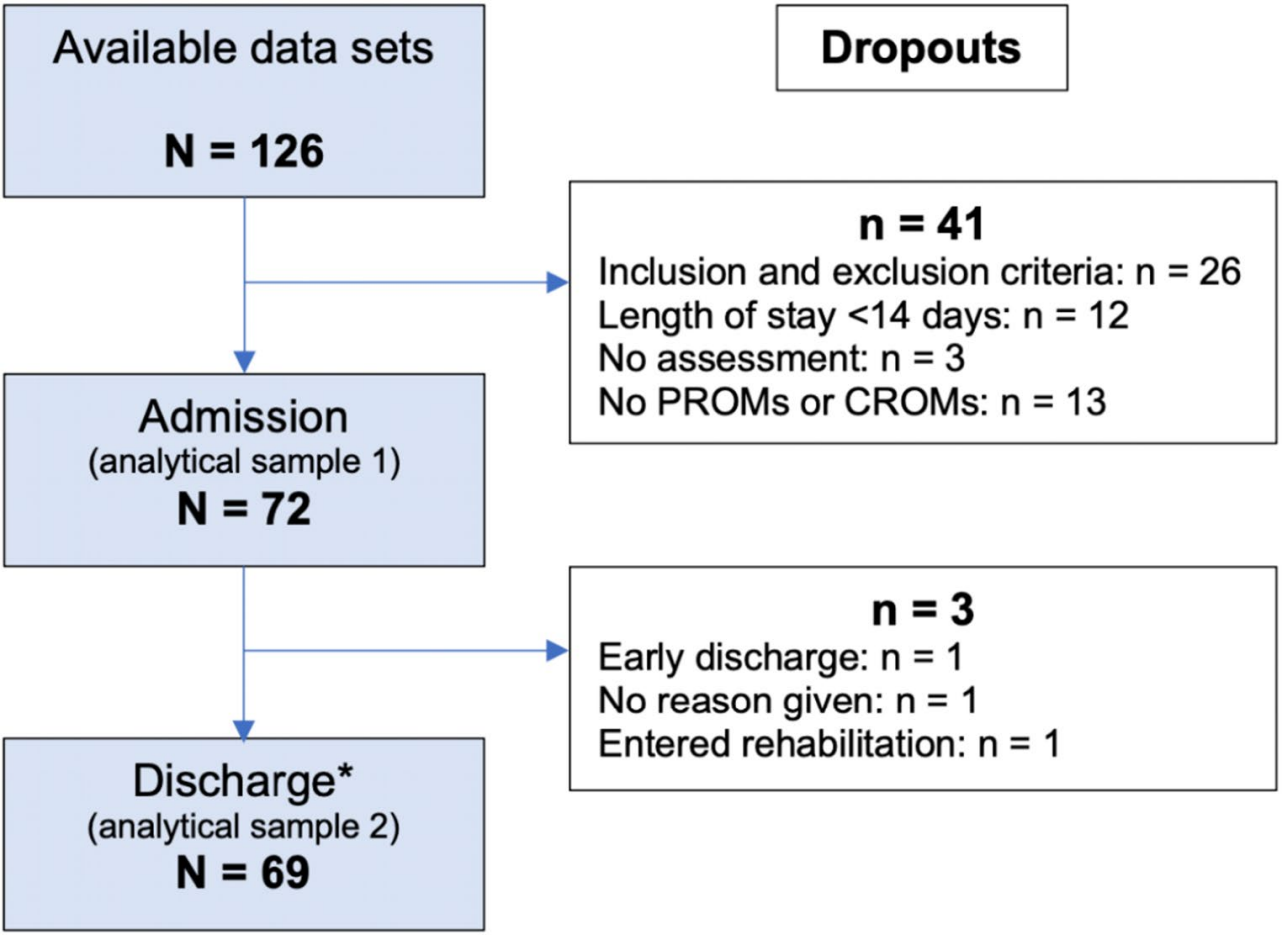
Study population from admission to discharge with dropouts. The data were analyzed according to protocol. *At discharge, 69 valid data sets were available, with missing individual data in the PROMs (*n* = 9) and CROMs (*n* = 7). Sarcopenia was calculated based on the CROMs, of which 62 values were available at discharge

Out of the 126 assessed transitional care patients, 26 patient datasets were excluded from the present analysis based on the inclusion and exclusion criteria. An additional 12 datasets were excluded because the patient stayed at the facility less than 14 days. Sixteen more datasets were excluded due to missing assessments, PROMs, or CROMs at admission. Thus, the final analysis included data from 72 transitional care patients at admission (analytical sample 1). A total of 69 valid data sets were available at discharge (analytical sample 2; Fig. 1). The included patients, all German-speaking, were referred from the district hospitals to transitional care.

At admission, 83.3% (*n* = 60) of the participants were female. On average, patients were 84 years old (age range: 60–97 years ± 6.64) with an average body mass index (BMI) of 23.7 (range: 13.6–38.9 ± 4.8). Half of the patients (*n* = 36) could be categorized as “underweight” (BMI < 23). The most common primary care indication were injuries of the lower extremities (44.4%, *n* = 32), followed by other, not primarily musculoskeletal, primary care indications (36.1%, *n* = 26). Ten patients (13.9%) showed impairments of the lumbar spine. Six patients (8.3%) were recorded as having an additional secondary care indication. Patients stayed for an average of 39 days (± 22 days). The patient demographics and characteristics are shown in Table 2.

**Table 2:** Demographic of patients in transitional care at admission

Patient Characteristics	Value (*N* = 72)
Age, mean (SD)	83.6 (± 6.64) years
Age, range	60–97 years
Length of stay (SD)	39 (± 22) days
Sex, *n* (%) *	
Female	60 (83.3%)
Male	12 (16.7%)
BMI, mean (SD)	23.68 (± 4.8) kg/m²
BMI, range	13.6–38.9
BMI category, *n* (%) *	
Underweight (< 23)	36 (50%)
Normal weight (23–29.9)	27 (37.5%)
Overweight (> 29.9)	8 (11.1%)
Primary care indication, *n* (%) *	
Lower extremities	32 (44.4%)
Upper extremities	3 (4.2%)
Spinal column^a^	11 (15.3%)
Basis^b^	26 (36.1%)

*p < .001 (χ^2^) = significant differences between groups. ^a^Spinal column includes the lumbar or the cervical spine. ^b^Basis = All other care indications that could not be assigned to a primarily musculoskeletal category (e.g., stroke, acute hepatitis, fungal pneumonia)

### Prevalence

#### Sarcopenia

Within the group classified as non- or pre-sarcopenic according to the SDOC criteria (*n* = 14), only one patient showed no indication of sarcopenia, while the remaining patients (*n* = 13) already fulfilled criteria corresponding to probable sarcopenia based on HGS (*n* = 4) or gait speed (*n* = 9). At admission, 80.6% of patients (*n* = 58) were sarcopenic. At discharge, 72.6% of patients (*n* = 45; *n* = 10 (13.9%) lost to follow-up due to missing CROMs) were sarcopenic. Over time, no significant changes in sarcopenia prevalence were observed (McNemar: *p* = .302, two-sided).

The prevalence of sarcopenia at admission and discharge was determined based on HGS and gait speed. During rehabilitation, HGS did not change significantly over time (*p* > .10), whereas gait speed increased from 28.44 ± 18.23 s at admission to 19.72 ± 9.23 s at discharge (*p* < .001).

Group comparisons (*t*-tests and χ^2^ tests) of sarcopenic vs. non- or pre-sarcopenic patients showed no differences in sociodemographic characteristics, namely age, sex, BMI and length of stay (*p* > .05). A trend (*p* < .10) was evident: Individuals with sarcopenia were on average older than those without sarcopenia (*M* = 84.6 ± 5.6 vs. *M* = 79.9 ± 9.1; *p* = .08; one-tailed: *p* = .04).

In the outcome measurements (EQ-5D-5 L, EQ-VAS, Barthel Index, NPRS, HAQ-DI, TUG, 10-MWT test, HGS asymmetry, MNA), only significant differences were found for the EQ-5D-5 L sum score and HGS asymmetry. At baseline, participants with sarcopenia reported significantly lower EQ-5D-5 L sum scores (*M* = 54.4 ± 17.5) than participants without sarcopenia (*M* = 65.71 ± 23.1; *p* < .05, *d* = 0.61). A trend was observed: Patients with sarcopenia demonstrated a higher HAQ-DI score (*M* = 1.87 ± 0.42) than patients without sarcopenia (*M* = 1.52 ± 0.61, *p* = .06; one-tailed: *p* = .03). Finally, a significantly higher proportion of participants with sarcopenia showed asymmetrical HGS (42 out of 56 patients) than those without sarcopenia (6 out of 14 patients). Overall, 68.6% of the total sample exhibited asymmetry. A χ^2^ test confirmed that a significant association existed between sarcopenia and HGS asymmetry (*p* = .02).

#### Malnutrition

At admission, 81.2% of patients (*n* = 56) were malnourished or at risk of malnutrition. To examine whether the prevalence or the risk of malnutrition was associated with demographic characteristics or relevant rehabilitation outcome assessments, group comparisons (*t*-tests and χ^2^ tests) were conducted at admission. Participants who were malnourished or at risk of malnutrition had a significantly lower BMI (*M* = 22.5 ± 4.9) than those without malnutrition (*M* = 28.1 ± 4.9, *p* < .001, *d* = 1.28). As for sarcopenia, no significant group differences (*p* > .10) were observed for any of the other variables between nutritional status and sociodemographic variables (age, sex), length of stay and outcome measurements (EQ-5D-5 L, EQ-VAS, Barthel Index, NPRS, HAQ-DI, TUG, 10-MWT, HGS).

A cross-tabulation was conducted to explore the overlap between sarcopenia and malnutrition. Among patients with sarcopenia, 80.4% were malnourished or at risk of malnutrition as compared to 84.6% of those without sarcopenia (Table 3). A χ^2^ test did not reveal a significant association between the presence of sarcopenia and nutritional status.

**Table 3:** Cross-tabulation of sarcopenia and malnutrition status at admission

	Malnutrition Risk (*n*, %)	No Malnutrition (*n*, %)	Total^b^
Sarcopenia	45 (80.4%)^a^	11 (19.6%)	56
No Sarcopenia	11 (84.6%)	2 (15.4%)	13
Total	56 (81.2%)	13 (18.8%)	69

Percentages refer to row totals. ^a^Corresponds to 65.2% of the total sample. ^b^*n* = 3 missing MNA out of *N* = 72

### Changes in rehabilitation outcomes

The patients’ quality of life [z values] increased significantly (*p* < .001) with mean scores ranging from *M* = -0.69 (± 1.1) at admission to *M* = 0.69 (± 0.87) at discharge, indicating a large effect (*d* = -1.41). Similarly, the patients’ care needs decreased significantly (*p* < .001) from admission (M = 0.54 ± 1) to discharge (M = -0.54 ± 1), also indicating a large effect (*d* = 1.09). Finally, mobility improved significantly (*p* < .001) in patients who had taken part in the transitional care program (*M* = -0.35 ± 1.26 to *M* = 0.35 ± 0.64), although the effect was moderate with a Cohen’s *d* = -0.56. Table 4 summarizes the results for each domain and corresponding outcome scales. Figure 3 in Appendix A illustrates the changes in outcome domains from admission to discharge.

**Table 4:** Changes in quality of life, care needs, and mobility from admission to discharge

Domain [z]	Subscale	Admission (M ± SD)	Discharge (M ± SD)	d (*p*)
Quality of Life	*Overall*	-0.69 ± 1.11	0.69 ± 0.87	-1.41*
	NPRS ^a^	3.73 ± 2.29	1.48 ± 1.27	1.05*
	EQ-5D-5 L ^b^	57.67 ± 19.12	73.43 ± 16.17	-0.84*
Care Needs	*Overall*	0.54 ± 0.99	-0.54 ± 1.02	1.09*
	Barthel Index	79.83 ± 11.46	90.39 ± 12.02	-0.83*
	HAQ ^c^	1.80 ± 0.46	1.31 ± 0.48	0.98*
Mobility	*Overall*	-0.35 ± 1.26	0.35 ± 0.64	-0.56*
	TUG ^d^	34.38 ± 17.61	24.14 ± 10.9	0.69*
	10-MWT ^e^	28.44 ± 18.23	19.72 ± 9.23	0.42*

All analyses were conducted using paired-sample t-tests comparing scores from admission to discharge. The table presents the z-standardized domain scores (Quality of Life, Care Needs, and Mobility) as well as their respective original subtest scores. For all outcomes, lower scores indicate improvement, except for the Barthel Index and EQ-5D-5 L, where higher scores reflect higher functional ability or quality of life, respectively* Indicates statistical significance at p < .001, two-sided^a^ NPRS = Numeric Pain Rating Scale. ^b^ EQ-5D-5 L = EuroQol 5-Dimension 5-Level Questionnaire. ^c^ HAQ = Health Assessment Questionnaire. ^d^ TUG = Timed Up and Go Test. ^e^ 10-MWT = 10-meter walking test

### Impact of sarcopenia

**Fig. 2:**
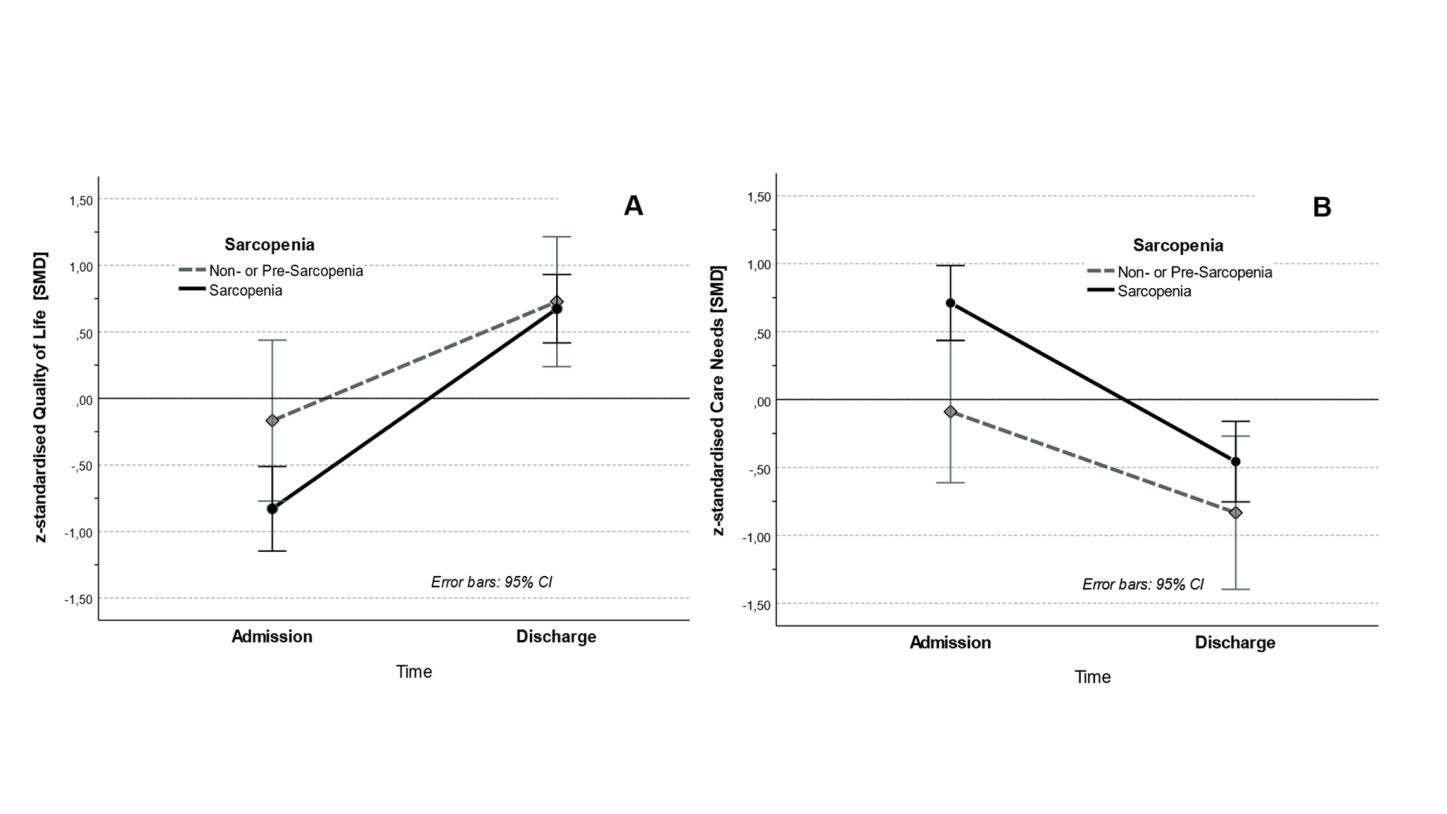
Time courses of quality of life (Fig. 2**A**) and care needs (Fig. 2**B**) of non- or pre-sarcopenic versus sarcopenic patients. Fig. 2**A** illustrates changes in patients’ quality of life from admission to discharge, showing that patients experienced a significant improvement over time and a significant interaction with sarcopenia status. This indicates a greater improvement in patients with sarcopenia. Figure 2**B** displays patients’ care needs over time, highlighting a significant reduction across the sample and higher overall care needs in patients with sarcopenia. Error bars represent standard errors. See main text for detailed statistics

**Fig. 3:**
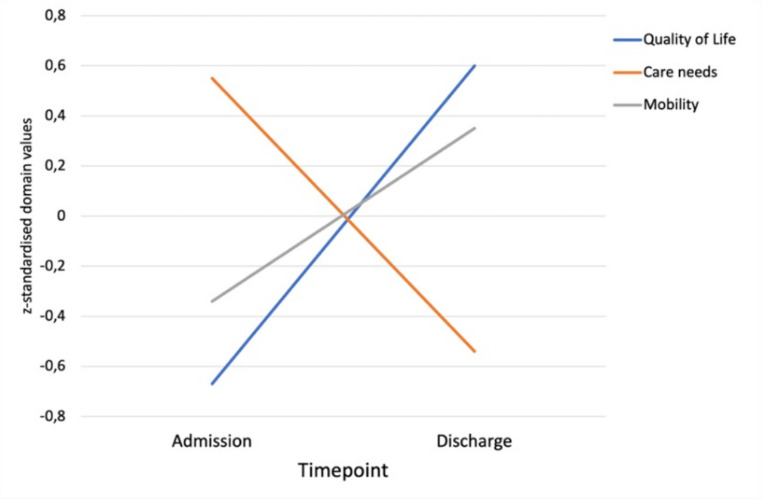
Changes in domains from admission to discharge. Figure displays changes in the patients’ quality of life, care needs, and mobility from admission to discharge. Scores are z-standardized (M = 0, SD = 1) and reflect improvements across all domains. Higher values indicate a better quality of life and mobility, while lower values indicate lower care needs. All changes were statistically significant (p < .001). See Table 4 for detailed statistics

A significant overall improvement in quality of life was observed in sarcopenic patients from admission to discharge (*p* < .001, η²ₚ = 0.529). Furthermore, a significant interaction between time and sarcopenia status was noted (*p* < .05, η²ₚ = 0.068). The trajectory of the quality of life differed depending on whether sarcopenia was present at admission or not. Patients with sarcopenia demonstrated greater improvements in quality of life during their rehabilitation stay compared to patients without sarcopenia (Fig. 2a). However, no significant main effect of the sarcopenia status on quality of life was observed *(p* = .194, η²ₚ = 0.029), indicating that the two groups did not experience a significantly different quality of life after rehabilitation.

The patients’ care needs decreased significantly over time (*p* < .001, η²ₚ = 0.401). Although the presence of sarcopenia had no influence on the change of care needs over time (*p* > .10, η²ₚ = 0.032), sarcopenia exhibited a significant main effect on care needs over time (*p* < .05, η²ₚ = 0.078). Patients with sarcopenia tended to have higher care needs than patients without (or pre-) sarcopenia (Fig. 2b).

Mobility increased significantly during the period of transitional care (*p* < .005, η²ₚ = 0.155). No interaction is noted between sarcopenia and mobility (*p* > .10, η²ₚ = 0.003), and no main effect of sarcopenia on mobility is seen (*p* > .10, η²ₚ = 0.019). However, patients with nutritional deficiencies tended to have higher overall mobility scores (*p* < .05, η²ₚ = 0.120). Nutritional status did not seem to exert an effect on the trajectory of quality of life or care needs. All results are reported in Table 5 in Appendix B.

## Discussion

The findings of our study highlight the high prevalence (> 65%) of sarcopenia and malnutrition in geriatric rehabilitation inpatients, supporting the need to conduct concomitant screening for muscle health and nutritional health in these geriatric care patients. Although the presence of sarcopenia and malnutrition at admission had no significant impact on the improvements observed during rehabilitation, sarcopenic patients still showed higher overall care needs at the end of the rehabilitation compared with patients who were in a non-or pre-sarcopenic state. Furthermore, our results demonstrate that these patients experience variable but often incomplete physical recovery after rehabilitation. This finding indicates that adequate follow-up and tailored interventions, such as progressive resistance training and nutritional support, are required during and after discharge to improve mobility and independence in terms of ADL in geriatric populations with nutritional deficiency and muscle weakness.

### Prevalence of sarcopenia and malnutrition

The prevalence of sarcopenia in this study population was 80.6%, while 81.2% of patients were malnourished or at risk of malnutrition. A co-occurrence of malnutrition and sarcopenia was observed in 65.2% of the enrolled patients, a proportion that was notably higher than in other geriatric rehabilitation studies with values ranging from 13.0% to 32.4% [8, 45, 46]. The high prevalence of sarcopenia observed in this cohort may be because the study only looked at a certain group of patients who needed rehabilitation and many of whom probably had sarcopenia. Furthermore, two-thirds of the current study’s participants with sarcopenia displayed asymmetrical HGS, which has been associated with functional limitations and increased odds for future falls [34, 47], and 71.6% of patients were discharged while still in a sarcopenic state.

The prevalence of sarcopenia (80.6%) in our cohort of geriatric patients admitted to a transitional care program was substantially higher than the prevalence found in two Swiss cohorts of geriatric rehabilitation patients (ranging from 22.6% to 40.2%) [45, 48] or the Australian RESORT study of geriatric rehabilitation patients (37.9%) [49]. While we defined sarcopenia according to the SDOC guidelines [33] as indicated by both reduced HGS and gait speed—thus not including a measure of skeletal muscle mass—the other studies identified patients according to the European Working Group on Sarcopenia in Older People (EWGSOP and EWGSOP2) guidelines [50, 51], including measures of muscle mass, strength, and function. The differences in the prevalence of sarcopenia between the studies may be partly explained by the lower HGS thresholds selected for the EWGSOP2 definition (< 16 kg for women and < 27 kg for men) compared with SDOC (< 20 kg for women and < 35.5 kg for men). However, in agreement with SDOC, the European Society for Clinical and Economic Aspects of Osteoporosis and Osteoarthritis (ESCEO) recommends that, in clinical trials of drugs aimed at treating sarcopenia, the target population should have a combination of low muscle strength and low physical performance, whereas low muscle mass has been shown to be less strongly correlated with health-related outcomes [52].

### Changes in proms and CROMs

Patients reported significant improvements in their quality of life, care needs, and mobility after taking part in the transitional care program, irrespective of the risk or diagnosis of malnutrition or sarcopenia. The measurement of PROMs, and especially of HRQoL measures, has shown that these are significant predictors of hard clinical outcomes, such as hospitalization or mortality [53], supporting the value of their use in the care of older adults. A recent meta-analysis combining 43 observational studies showed a significant decrease in HRQoL in sarcopenic compared with non-sarcopenic older people [13]. In our cohort, quality of life was reduced in patients with sarcopenia at admission, but sarcopenic patients showed a greater improvement over time than patients without sarcopenia (*p* < .001). The use of disease-specific HRQoL assessment tools, however, such as the specific Sarcopenia and Quality of Life questionnaire (SarQoL) [54], may enable researchers to more clearly discriminate between sarcopenic patients with respect to their quality of life.

In the present study, the Barthel Index and the Health Assessment Questionnaire (HAQ-DI) were used to assess patients’ care needs. We observed a significant reduction of overall care needs in the sample at the end of the rehabilitation (*p* < .05), although these were still higher in patients with sarcopenia than in patients without sarcopenia. Although the majority (87.2%) of patients were discharged to return to and remain at home after three months, many continued to experience functional limitations and pain. This highlights the importance of providing ongoing rehabilitation and care support services.

During rehabilitation, the HGS of the patients in the current study did not change (*p* > .10), but their physical performance improved on average, namely their gait speed, namely their time required to walk 10 m (from 28.44 ± 18.23 s at admission to 19.72 ± 9.23 s at discharge, *p* < .001 and TUG score (ranging from 34.38 ± 17.61 s at admission to 24.14 ± 10.00 s at discharge, *p* < .001).

Our study showed no change in HGS during rehabilitation which indicates that the program could not reverse sarcopenia even after successful rehabilitation. The lack of improvement in HGS further suggests that HGS alone may not always reflect lower body function, thus directly capture the more specific lower body functional impairments that influence physical performance [55]. The Swiss study conducted in 5 rehabilitation centers with different disciplines of inpatient rehabilitation also reported a substantial number of patients with low values of HGS at discharge [45]. HGS may not improve after rehabilitation if the program was too short or if other factors, such as continued muscle damage, comorbidities, malnutrition and inflammation are at play. Thus, standard treatment of the underlying illness and classic physical therapy may not sufficient to normalize the skeletal muscle strength in these geriatric rehabilitation patients [56].

Gait speed has been shown to be highly predictive of future care dependence, severe mobility limitations, and mortality [57, 58]. The clinically meaningful improvements have been estimated at 0.05–0.1 m/s [59]. The TUG is a proposed measurement tool by the EWGSOP2 [51] for muscle function that has been recently used to predict hospitalization-associated functional decline in older adults [60]. The latter findings demonstrate that hospitalized older adults unable to successfully complete the TUG test under 12 s showed significant functional decline after hospitalization. Thus, early mobilization intervention efforts immediately post-discharge are needed to counteract the negative impacts of acute hospitalization. Here, a transitional care program could play a crucial role.

### Strengths and limitations

A major strength of this study is the inclusion of old and very old subjects and the use of validated PROMs and CROMs that can be applied in clinical settings by various stakeholders. However, several limitations must be considered when interpreting the data. This single-site study had a small sample size due to its exploratory study design, which could limit generalizability of the findings to other geriatric rehabilitation settings. In addition, the study did not include all older adults who were hospitalized, but rather those requiring rehabilitation, namely, individuals with reduced physical function or ADL, many of whom were likely affected by sarcopenia. As a result, the high prevalence of sarcopenia and malnutrition observed in this cohort may further limit the generalizability of our findings. Although the EWGSOP2 criteria would allow for more meaningful comparisons with prior studies, the SDOC criteria, which does not include muscle mass, were chosen to identify patients with sarcopenia because of their high discriminatory accuracy for identifying people with disability. However, the Society on Cachexia and Wasting Disorders (SCWD) recommends the use of HGS to measure muscle strength and the use of validated measures of physical performance (e.g., the Short Physical Performance Battery or habitual gait speed tests) for the assessment of muscle weakness and physical performance in daily clinical practice, where the focus shifts to functional limitations and disability outcomes [61]. Furthermore, relevant covariates such as the patients’ dietary intakes or the exercise type or intensity were not assessed. For practical reasons, we also did not take CROMs at follow-up (via telephone interview), which would be necessary to consider when assessing the program’s success and sustainability. Finally, the variability in program duration reflects real-world clinical practice, but it may have introduced heterogeneity in participants’ exposure to the intervention. This should be considered when interpreting the observed outcomes.

## Conclusions and implications

The prevalence of sarcopenia in geriatric rehabilitation inpatients admitted to a transitional care program was high and associated with the patients’ lower functional ability to perform ADL, higher overall care needs, and reduced quality of life compared with inpatients without sarcopenia. These findings emphasize the importance of interventions tailored to mitigate sarcopenia, preferably multicomponent exercise training sessions (placing a focus on resistance training at least twice a week) combined with nutritional support (placing a focus on a protein intake above the RDA) [62, 63]. Furthermore, malnutrition was highly prevalent in the geriatric patients undergoing inpatient rehabilitation in our study. Transitional care successfully improved rehabilitation outcomes, but not HGS (*p* > .05). The observed high prevalence of sarcopenia and malnutrition in geriatric rehabilitation patients highlights the need for enhanced rehabilitation programs with follow-up care, offering early screening for sarcopenia and malnutrition, to ensure that older adults are assessed and treated accordingly. Further research with a larger sample size is needed.

## Data Availability

The research data supporting this publication are stored in our institutional digital data repository for published research, accessible via [https://creed.lbg.ac.at](https:/creed.lbg.ac.at) (on 21 October 2025). The data sets analyzed in this manuscript are not publicly available due to ethical and legal restrictions, as they contain potentially identifying and sensitive patient information. However, pseudonymized data sets have been created for the purpose of re-use and are also accessible at creed.lbg.ac.at. Requests for access to anonymized data sets should be directed to the corresponding author (V.G.).

## Appendix A

See Fig. 3.

## Appendix B

See Table 5.

**Table 5:** Changes in quality of life, care needs, and mobility from admission to discharge depending on sarcopenia status

Domain ^g^	Subscale	Sarcopenia Status	Admission (M ± SD)	Discharge (M ± SD)
Quality of Life	Overall ^f^	Non- or Pre-sarcopenic	-0.26 (± 1.22)	0.73 (± 0.94)
		Sarcopenic	-0.78 (± -0.69)	0.67 (± 0.73)
	NPRS ^a^	Non- or Pre-sarcopenic	3.36 (± 1.78)	1.69 (± 1.6)
		Sarcopenic	3.66 (± 2.31)	1.43 (± 1.17)
	EQ-5D-5 L ^b^	Non- or Pre-sarcopenic	65.71 (± 23.11)	76.54 (± 15.60)
		Sarcopenic	54.40 (± 17.55)	72.55 (± 16.38)
Care needs	Overall ^f^	Non- or Pre-sarcopenic	0.03 (± 1.22)	-0.83 (± 0.91)
		Sarcopenic	0.68 (± 0.92)	-0.46 (± 1.04)
	Barthel Index	Non- or Pre-sarcopenic	83.57 (± 12.77)	93.52 (± 7.67)
		Sarcopenic	78.62 (± 11.27)	89.52 (± 12.90)
	HAQ ^c^	Non- or Pre-sarcopenic	1.69 (± 0.62)	1.38 (± 0.57)
		Sarcopenic	1.87 (± 0.42)	1.34 (± 0.45)
Mobility	Overall ^f^	Non- or Pre-sarcopenic	-0.07 (± 1.39)	0.49 (± 0.60)
		Sarcopenic	-0.41 (± 1.15)	0.32 (± 0.65)
	TUG ^d^	Non- or Pre-sarcopenic	31.89 (± 20.65)	20.80 (± 10.58)
		Sarcopenic	35.33 (± 15.81)	24.69 (± 11.07)
	10-MWT ^e^	Non- or Pre-sarcopenic	23.96 (± 20.62)	18.79 (± 9.67)
		Sarcopenic	28.99 (± 16.55)	19.97 (± 9.20)

All analyses were conducted using paired-sample t-tests comparing scores from admission to discharge. The table presents the z-standardized domain scores (Quality of Life, Care Needs, and Mobility) as well as their respective original subtests. For all outcomes, lower scores indicate improvement, except for the Barthel Index and EQ-5D-5 L, where higher scores reflect higher functional ability or quality of life, respectively.* indicates statistical significance at p < .01, two-sided. ** indicates stat. sign. at p < .001, two-sided^a^ NPRS = Numeric Pain Rating Scale. ^b^ EQ-5D-5 L = EuroQol 5-Dimension 5-Level Questionnaire. ^c^ HAQ = Health Assessment Questionnaire. ^d^ TUG = Timed Up and Go Test. ^e^ 10-MWT = 10-meter walking test. ^f^ Univariate 2 × 2 (Time [2], Sarcopenia [2]) for each domain: between effect (sarcopenia status) as indicated by Quality of Life p = .194, η²ₚ = 0.029, Care needs p < .05, η²ₚ = 0.078), and Mobility p > .10. η²ₚ = = 0.019. Interaction effect (Time x Sarcopenia) as indicated by Quality of Life (p < .05, η²ₚ = 0.068), Care needs p > .10, η²ₚ = 032, and Mobility p > .10, η²ₚ = 0.0003. ^g^ An overall rehabilitation outcome was computed by calculating the mean of the z-standardized domain scores (Quality of Life, Care Needs, and Mobility) and then z-standardizing the data. The analysis was conducted using paired-sample t-test, comparing scores from admission to discharge. From admission to discharge, an overall improvement in rehabilitation outcomes was observed (d = 0.68, p < .01)
